# The Influence of Visual Input on Electromyographic Patterns of Masticatory and Cervical Spine Muscles in Subjects with Myopia

**DOI:** 10.3390/jcm10225376

**Published:** 2021-11-18

**Authors:** Grzegorz Zieliński, Anna Matysik-Woźniak, Maria Rapa, Michał Baszczowski, Michał Ginszt, Magdalena Zawadka, Jacek Szkutnik, Robert Rejdak, Piotr Gawda

**Affiliations:** 1 Department of Sports Medicine, Medical University of Lublin, 20-093 Lublin, Poland; m.baszczowski@gmail.com (M.B.); magdalena.zawadka@umlub.pl (M.Z.); piotr.gawda@umlub.pl (P.G.); 2 Department of General and Pediatric Ophthalmology, Medical University of Lublin, 20-093 Lublin, Poland; anna.wozniak@umlub.pl (A.M.-W.); robert.rejdak@umlub.pl (R.R.); 3 Students’ Scientific Association at the Department of General and Pediatric Ophthalmology, Medical University of Lublin, 20-093 Lublin, Poland; maria.rapa00@gmail.com; 4 Department of Rehabilitation and Physiotherapy, Medical University of Lublin, 20-093 Lublin, Poland; michal.ginszt@umlub.pl; 5 Department of Functional Masticatory Disorders, Medical University of Lublin, 20-093 Lublin, Poland; jacek.szkutnik@umlub.pl

**Keywords:** myopia, electromyography, masticatory muscles, activity index, asymmetry index, functional indices

## Abstract

This study aimed to analyze the change of visual input on electromyographic patterns of masticatory and cervical spine muscles in subjects with myopia. After applying the inclusion criteria, 50 subjects (18 males and 32 females) with myopia ranging from −0.5 to −5.75 Diopters (D), were included in the study. Four muscle pairs were analyzed: the anterior part of the temporalis muscle (TA), the superficial part of the masseter muscle (MM), the anterior belly of the digastric muscle (DA), and the middle part of the sternocleidomastoid muscle belly (SCM) during resting and functional activity. Statistical analysis showed a significant decrease within functional indices (FCI) for the sternocleidomastoid muscle (FCI SCM R, FCI SCM L, FCI SCM total) during clenching in the intercuspal position with eyes closed compared to eyes open. During maximum mouth opening, a statistically significant increase of functional opening index for the left temporalis muscle (FOI TA L) was observed. Within the activity index (AcI), there was a statistically significant decrease during clenching on dental cotton rollers with eyes closed compared to eyes open.

## 1. Introduction

Myopia is a common condition that develops primarily in childhood and early adulthood where the optical system of the eye focuses light rays incorrectly [1]. Myopia is one of the most common eye diseases in the world. It is estimated that 1.4 billion people had myopia in 2000 and this number is expected to reach 4. 8 billion by 2050 [2]. High prevalence rates pose a major public health challenge due to visual impairment [3]. The global potential productivity loss associated with the burden of visual impairment in 2015 was estimated at 244 billion dollars due to uncorrected myopia [4]. It is well known that myopia is associated with several ocular complications such as retinal detachment, glaucoma, cataract, optic disc changes, and maculopathy [5]. Complications related to other systems are increasingly recognized.

Observations linking the stomatognathic system to the organ of vision have also been made; for example, myopia is more common in individuals with second class 1st division, while astigmatism is more characteristic in individuals with crossbite [6]. Some authors have suggested that individuals with refractive and oculomotor disorders are more affected by headaches than healthy individuals [7,8]. The stomatognathic system is a functional complex of tissues and organs located within the oral and craniofacial cavities [9]. 

The neurological connection between to the organ of vision and stomatognathic systems would hypothetically be associated with the vestibulo-ocular reflex (VOR). The VOR keeps us balanced even though our eyes and head are constantly moving. When we make a head movement, our eye muscles are immediately activated, and they make the eye movement opposite to the head movement at the same speed. The VOR adjusts the visual field, this combines with retinal image stabilization to keep the eye in space, and focused on the object despite head movements [10]. A reflex that further assists in achieving stabilization of the visual target and image on the retina is the cervico-ocular reflex, which works in conjunction with the vestibulo-ocular reflex. The vestibular complex receives afferent information from other parts of the central nervous system, including the spinal cord, cerebellum, and parts of the midbrain nuclei, reticular formation [11]. The reticular formation also plays an important role in gaze, head movements, and saccadic coordination. Different parts of the reticular formation are responsible for different functions of the eye [12]. It is noteworthy that trigeminal neurons project directly to the nuclei of the reticular formation and exhibit the response to afferent stimuli through reflex movements. Stimulation of the reticular formation nuclei can lead to increased muscle tone and muscle spasms, e. g., causing facial and neck muscle twitching [13,14]. People with myopia who do not use correction or use the wrong correction (corrective glasses, lenses) may experience stimulation of the described pathways and increased activity of the masticatory organ and neck muscles. It has been noted that people suffering from refractive and oculomotor disorders are more affected by headaches than healthy people, which may be associated with an increase in the activity of the temporalis muscles [7,8]. Sustained increased bioelectrical activity may lead to changes in the electromyographic patterns of the masticatory muscles, causing, e.g., temporomandibular disorders (TMDs) or tension-type headache (TTH) [15]. 

On the basis of clinical observations, there was made a correlation between the masticatory muscles and the organ of vision [7]. Observed changes in the bioelectrical activity of the masticatory muscles during changes in the visual stimulus (eyes open versus eyes closed) in patients with myopia have been reported [16]. Changes in muscle bioelectrical activities during visual stimulus changes have also been observed in individuals with TMDs [7,17]. However, in the study on people without visual impairment, no difference was found within masticatory muscle activity between closed and open eyes measurements [18].

The purpose of this study was to analyze the change of visual input on electromyographic patterns of masticatory and cervical spine muscles in subjects with myopia. It was hypothesized that the visual input would influence electromyographic patterns of masticatory and cervical spine muscles in subjects with myopia. To the best of our knowledge, this is the first study analyzing changes in bioelectric activity, asymmetry, and functional indices of masticatory and cervical spine muscles in myopic subjects.

## 2. Materials and Methods

### 2.1. Study Population

Seventy-nine individuals with myopia were invited to participate in the study. The study was conducted in compliance with the Declaration of Helsinki and was approved by the local Bioethics Committee of the Medical University of Lublin (approval number KE-0254/229/2020). Participants were informed of the objectives of the study and were aware of their ability to opt-out at any time. Written consent was obtained from all respondents who participated in the study.

The inclusion criteria used in the study were: myopia based on clinical examination, four zones of arch support, complete dentition. The following exclusion criteria were used in the clinical examination: hyperopia, ocular diseases, optic nerve diseases, TMDs symptoms based on The Research Diagnostic Criteria for Temporomandibular Disorders (RDC/TMD) examination, class II and III according to Angle’s classification, open bite, crossbite, inflammatory conditions within the oral cavity, neurological disorders in the head and neck region, neoplastic diseases (regardless of type and location), neck and shoulder pain regardless of etiology within the last 6 months, trauma and previous surgical treatment in the head and neck region within the last 6 months of examination, pregnancy.

Two clinical examinations were conducted to qualify for the present experiment. The first was a clinical trial based on the RDC/TMD dual-axis questionnaire. It was conducted by an experienced dentist with a specialization in dental prosthetics (author J. S.). Clinical evaluation of RDC/TMD was performed using standardized palpation of the temporomandibular joints, and surrounding tissues, according to RDC/TMD guidelines [19]. The RDC/TMD examination was extended to include palpation and functional examination of the cervical spine muscles and associated structures (cervical spine joints and ligaments) by a physiotherapist specializing in neck therapy (author G. Z.).

The second clinical examination evaluated the structures of the eyeball using a slit lamp. Subjects with known ocular diseases were removed from the study. It was conducted by an experienced medical doctor specializing in eye diseases (author A. M-W.).

After applying exclusion criteria, 50 subjects (18 men and 32 women) were qualified for the study.

### 2.2. Study Protocol

#### 2.2.1. Assessment of the Muscle Activity

The sEMG examination was performed using a BioEMG III 8-channel electromyograph, compatible with the BioPAK measurement system (BioResearch Associates, Inc., Milwaukee, WI, USA). The sEMG studies were conducted between 8:00 am and 12:00 pm to minimize the influence of diurnal fluctuations on muscle activity. Four muscle pairs were analyzed: the anterior part of the temporalis muscle (TA), the superficial part of the masseter muscle (MM), the anterior belly of the digastric muscle (DA), and the middle part of the sternocleidomastoid muscle belly (SCM).

**Figure 1:**
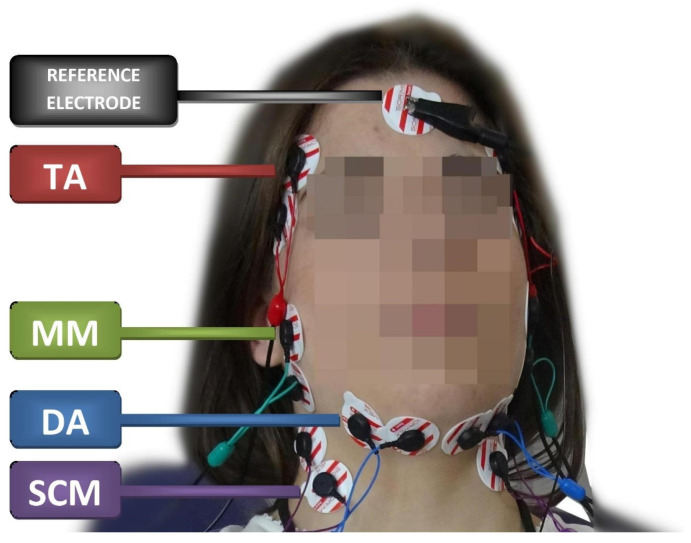
Electrode placement during the electromyographic examination. TA—the temporalis anterior; MM—the superficial part of the masseter muscle; DA—the anterior belly of the digastric muscle; SCM—the middle part of the sternocleidomastoid muscle belly.

The subjects were seated in the standardized position on the dental chair; the height of the head restraint was adjusted individually to align the head, neck, and torso of the subjects. Before placing surface electrodes (Ag/AgCl with a diameter of 30 mm and a conductive surface of 16 mm—SORIMEX, Torun, Poland), the skin was disinfected with 90% ethanol. The electrodes were symmetrically placed on the skin covering the examined muscles on both sides according to the course of the muscle fibers preceded by palpation of the muscles during mandibular movements. The edges of the electrodes covering the skin over a given muscle were in contact with each other to maintain equal electrode spacing. The reference electrode was placed on the forehead where there is no typical muscle tissue under the skin [20]. The electrode was placed according to the SENIAM standards [21] (Figure 1). Placing surface electrodes was performed by the same physiotherapist (author G. Z.).

Electromyographic activity was recorded in four conditions: at rest (10 s), during maximal voluntary clenching in the intercuspal position (as hard as possible; 3 × 3 s, 2 s rest between), during maximal voluntary clenching on dental rollers (as hard as possible; 3 × 3 s, 2 s rest between), and during maximal mouth opening (as wide as possible; 3 × 3 s, 2 s rest between). Averaged records from three trials were included in the analysis [22]. It was conducted with open as well as closed eyes with a 5 min break between tests. There was a random selection of the initial test. Recording the sEMG signal was performed without visual correction in the form of glasses and lenses. Subjects looked ahead in the open eye test [7,16]. Before each test, an interference test was performed using BioPAK Measurement System (BioResearch Associates, Inc. Milwaukee, WI, USA). In addition, BioPAK Noise Tests were administered to all participants after each measurement. Electromyographic signals obtained during the test were amplified with a minimum noise up to 5000 times stronger than their original level and cleaned of 99% linear scale noise with a frequency higher than 50/60 Hz from the data registered during the analysis using a digital BioPAK NoiseBuster filter. The recorded signal was filtered through a band bass filter between 20 and 400 Hz. The automatic processing of the electromyographic signal based on root means square (RMS) calculation in the BioPAK program allowed us to obtain the average measurement of values, which were then used for the analysis of muscle activity. Moreover, all the electromyographic signals were verified visually before each RMS calculation to obtain RMS only over steady state periods. The sEMG protocol was tested by dual sEMG measurements on 10 participants. These two independent sEMG measurements were separated by 5 min of rest between activities [23]. There were no significant differences between repeated sEMG recordings in all variables analyzed in the mandibular resting position. The signal analysis was performed by the same physiotherapist (author G. Z.).

#### 2.2.2. Ophthalmic Examination

The current gold standard for measuring visual acuity in clinical practice and research has been used. That is the back-lit logarithm of the minimum angle of resolution (logMAR) and it was used in the Early Treatment Diabetic Retinopathy Study (ETDRS) [24]. Visual acuity testing using the ETDRS card was performed from a distance of 4 m. Each line of the graph contains five optotypes, and their size varies by a constant proportion in each line [24]. This study was conducted by an experienced medical doctor specializing in eye diseases (author A. M.-W.). The results of the ETDRS array were confirmed in a Topcon KR-800 autokeratorefractometer test (Topcon Co. Tokyo, Japan). It is recognized as a rapid and accurate option for ocular screening [25]. According to the latest recommendations (2019), myopic individuals were defined as those with a refractive error ≤−0.50 diopters (D); these were subjects with low myopia [26].

The study group included subjects with a defect of −0.5 D to −5.75 D. The mean defect value was −2 D (±1.5 D) for the right eye and −2 D (±1.5 D) for the left eye. 

#### 2.2.3. Activity and Asymmetry Indexes 

The activity index (AcI) and asymmetry index (AsI) were counted from the RMS. AcI values range between +100% and −100%, with +100% indicating the involvement of only the masseter muscle during activity and −100% of only the temporalis muscle [27]. This index was proposed by Naeije et al.; the formula is as follows:

Activity index (AcI) = (RMS_MM_ − RMS_TA_)/(RMS_MM_ + RMS_TA_) × 100 (1)

to assess asymmetry in the activity of the muscles of the masticatory organ of the right and left side of the AsI. Its values range between +100% and −100%, with +100%, indicating the involvement of the tested muscles during activity on the right side only, −100% on the left side only. On the other hand, a value of 0% of the asymmetry index defines the equal activity of the tested muscles on the right and left sides [27]. This index was proposed by Naeije et al.; the formula is as follows:

Asymmetry Index (AsI) = (RMS_right_ − RMS_left_)/(RMS_right_ + RMS_left_) × 100 (2)

#### 2.2.4. Functional Indices

Functional Clenching (FCI) and Functional Opening (FOI) indices were obtained as the ratio of the difference between the mean muscle RMS potentials during activity, including clenching (CL) and opening (MMO) and the mean resting (REST) potentials, were proposed by Ginszt and Zieliński [28] using the following formulas:

Functional Clenching Index for TA right or left-sided (FCI_TA-R or L_) = CL_TA-R or L_/REST_TA-R or L_ (3)

Functional Clenching Index for TA both-sided (FCI_TA_) = (CL_TA-R_ + CL_TA-L_)/(REST_TA-R_ + REST_TA-L_) (4)

Functional Clenching Index for MM right or left-sided (FCI_MM-R or L_) = CL_MM-R_/REST_MM-R_ (5)

Functional Clenching Index for MM both-sided (FCI_MM_) = (CL_MM-R_ + CL_MM-L_)/(REST_MM-R_ + REST_MM-L_) (6)

Functional Clenching Index for SCM right or left-sided (FCI_SCM-R or L_) = CL_SCM-R or L_/REST_SCM-R or L_ (7)

Functional Clenching Index for SCM both-sided (FCI_SCM_) = (CL_SCM-R_ + CL_SCM-L_)/(REST_SCM-R_ + REST_SCM-L_) (8)

Functional Opening Index right or left-sided (FOI_R or L_) = MMO_DA-R or L_/REST_DA-R or L_ (9)

Functional Opening Index both-sided (FOI) = (MMO_DA-R_ + MMO_DA-L_)/(REST_DA-R_ + REST_DA-L_) (10)

Next, based on FCI and FOI indices, the following formulas were used for the assessment Functional Clenching Activity Index (FCAI), Functional Clenching Symmetry Index (FCSI), and Functional Opening Symmetry Index (FOSI) [28]:

Functional Clenching Activity Index right or left -sided (FCAIR) = (FCI_MM-R or L_ −FCI_TA-R or L_)/(FCI_MM-R or L_ + FCI_TA-R or L_) × 100 (11)

Functional Clenching Activity Index both-sided (FCAI) = (FCI_MM_ −FCI_TA_)/(FCI_MM_ + FCI_TA_) × 100 (12)

Functional Clenching Symmetry Index for TA (FCSI_TA_) = (FCI_TA-R_ −FCI_TA-L_)/(FCI_TA-R_ + FCI_TA-L_) × 100 (13)

Functional Clenching Symmetry Index for MM (FCSI_MM_) = (FCI_MM-R_ − FCI_MM-L_)/(FCI_MM-R_ + FCI_MM-L_) × 100 (14)

Functional Clenching Symmetry Index for SCM (FCSI_SCM_) = (FCI_SCM-R_ − FCI_SCM-L_)/(FCI_SCM-R_ + FCI_SCM-L_) × 100 (15)

Functional Clenching Symmetry Index for DA (FCSI_DA_) = (FCI_DA-R_ − FCI_DA-L_)/(FCI_DA-R_ + FCI_DA-L_) × 100 (16)

Functional Opening Symmetry Index (FOSI) = (FOI_R_ − FOI_L_)/(FOI_R_ + FOI_L_) × 100 (17)

Functional Opening Activity Index (FOAI) = (FOI_R_ − FOI_L_)/(FOI_R_ + FOI_L_) × 100 (18)

The functional indices allow to determine the presence of asymmetry in the activity of the masticatory muscles while taking into account the functional and resting activity. The disturbance of the proportion between resting and functional activity may indicate an imbalance within the masticatory organ, which has been observed in patients with TMDs. The decrease in functional indices’ values may be associated with an abnormal/compensatory pattern of masticatory muscle activity [28].

#### 2.2.5. Statistical Analysis

These calculations were performed using the Statistica™ version 13.3 (TIBCO Software Inc., Palo Alto, CA, USA). Normality of data distribution was tested by Shapiro–Wilk test. Student’s paired t-test was used for comparisons of two dependent groups when assumption of normal distribution was met and the Wilcoxon matched-pairs test for paired samples was used for data, which showed no compatibility with normal distribution. The a priori alpha level was set at *p* ≤ 0.05. The Chi-square test was used to compare the number of females and males in groups. To compare groups, the Mann–Whitney U-test was used. Effect sizes were determined for *t*-test using the Cohen d method and interpreted as small (0.2), medium (0.5), and large (0.8) effect sizes. For the Wilcoxon Z-test, Cohen’s guidelines for r are that a large effect is 0.5, a medium effect is 0.3, and a small effect is 0.1 [29,30,31].

An analysis of power was conducted using G*Power 3.1 [32]. The calculations indicated that a sample size of 44 participants would be sufficient to notice a significant differences between matched pairs (*t*-test) with an *α* value of 0.05, a power value of 0.90, and an estimated medium effect size of 0.50.

## 3. Results

There was a statistically significant decrease in AcI total values during closed eyes in comparison to open eyes during clenching on dental cotton rollers (Table 1). In terms of other indices, the differences between the two conditions did not reach the assumed significance level.

Based on the statistical analysis, significantly lower values of functional indices were observed during eyes closed in comparison to eyes open within FCI SCM R, FCI SCM L, and FCI SCM total, as presented in Table 2. During maximum mouth opening, a statistically significant increase of FOI TA L value was observed with eyes closed in comparison to eyes open. In terms of other indices, the differences between the two conditions did not reach the assumed significance level (Table 2).

## 4. Discussion

Masticatory muscle activity is controlled by the trigeminal nerve and information flowing through neuromuscular spindles that provide proprioceptive information [33]. Proprioception relies on populations of mechanosensory neurons distributed throughout the body which are collectively referred to as proprioceptors. They are specifically located within muscles and joints [34]. These inputs play an important role in the maintenance and modifications of the muscle basal tone [33,35]. Afferent impulses from proprioceptors cooperate with labyrinthine impulses to support oculomotor muscle activity through the corticothalamic–vestibulo-ocular reflex (VOR) [16]. VOR starts to work when a head movement is performed. The eye muscles are immediately triggered to induce eye movement opposite to the movement of the head at the same speed to adjust the visual world, which in turn stabilizes the image of the retina. It keeps the eye still in space and focused on the object, despite the movement of the head [10]. When there is no visual stimulus (eyes closed) and lack of movement (standardized position), there is a decrease in the bioelectrical activity of selected muscles [16]. Marchili et al. indicated neurophysiological connections in the form of the nucleus of the intermediate medulla which connects information from the head and neck and relays it onto the nucleus of the solitary tract. In this tract, autonomic responses are generated. Its task is to integrate information from the head and neck and transmit it to the nucleus of the solitary pathway, where autonomic responses are generated [36].

This study was conducted to analyze the change of visual input electromyographic patterns of masticatory and cervical spine muscles on subjects with myopia. Statistical analysis showed no significant changes in AsI and AcI during Rest, Clenching, and maximum mouth opening. There was a statistically significant decrease in AcI values during closed eyes test in comparison to open eyes test during clenching on dental cotton rollers. Statistical analysis showed significant statistical differences in the decrease in tensor activity of closed eyes in comparison to open eyes in FCI SCM R, FCI SCM L, and FCI SCM total during clenching in the intercuspal position. Statistical analysis showed no changes in functional indices during clenching on dental cotton rollers. During maximum mouth opening, a statistically significant increase of FOI TA L values was observed.

According to a study conducted by Monaco et al. evaluating 10 children with myopia aged 7–13 years, a decrease in activity on temporal muscles in a closed-eye test was seen [16]. The study was conducted by Ciavarella et al. (*n* = 28 aged 16–48 years) on myopic subjects with myofascial pain that showed large changes in bioelectrical activities within the TA and MM during a change in visual stimulus (eyes open versus eyes closed). A decrease in activity was demonstrated during the closed eye test [17]. In our study, we did not analyze bioelectrical changes, but changes within the indices (AcI, AsI and Functional Index). Changes in electromyographic indices within the TA and SCM were observed. This partly confirms the findings of Monaco et al. [16] and Ciavarella et al. [17].

Patients often compensate for vision problems by leaning forward or turning their head from side to side. These individuals often have a protracted position of the head and cervical spine, which leads to increased tension of the thoracic muscles, descending fibers of the quadriceps, scapular lever, sternoclavicular and mastoid muscles, and suboccipital muscles [37]. Long-term shortening of the above-mentioned muscles may cause ischemia, formation of musculofascial trigger points (MTrPs), which may result in pain, e. g., dizziness, tinnitus, and neck stiffness [38]. It has been demonstrated that both the presence of MTrPs in the upper trapezius and TMD are associated with changes in the electromyographic patterns of the masticatory muscles [22]. Muscular asymmetry was indicated as one of the factors predisposing to TMDs [15]. Based on the above studies, myopia can be suggested as a predictor of muscle imbalance, causing greater involvement of the TA and SCM muscles [37].

Our results seem to confirm the connections between temporalis anterior and the organ of vision. Increased electromyographic indices on the TA during open-eye in myopia might cause tension-type headaches because of the lack of correction or inadequate correction. These studies show an important role of the visual input in changes in the activity of the masticatory muscles, which should be taken into consideration during the diagnosis and therapy of patients with myopia.

The study presented here has several limitations. Firstly, the diagnostic criteria for TMDs were changed to The Diagnostic Criteria for Temporomandibular Disorders (DC/TMDs) in 2014. However, in the presented study, the previous version was used. So far, there is no validated Polish version of DC/TMD; therefore RDC/TMD was used. Secondly, in presented study, we only studied people with myopia. Hence, a comparison of the results of myopia patients with those of the healthy population would be advisable in subsequent studies.

## 5. Conclusions

Closed eyes during sEMG examination are associated with a decrease in activity index values during clenching on dental cotton rollers, with a decrease within all functional clenching indices for sternocleidomastoid muscle during clenching in the intercuspal position, and with an increase of functional opening index within left temporalis muscle, in comparison to open eyes test. Thus, closing and opening eyes in patients with myopia may be associated with a change in electromyographic patterns within cervical and masticatory muscles. Further research may explain the influence of visual input on masticatory muscles activity in patients with myopia.

## Figures and Tables

**Table 1:** The comparison of the results of the activity index and asymmetry during test (eyes open and closed) at rest, clenching, clenching on dental cotton rollers, and maximum mouth opening.

	Indices	Eyes Open				Eyes Closed				Statistics		
		M	SD	95%CI		M	SD	95%CI		Test	Test Result	*p* Value
Rest	AsI TA	−6.66	23.72	−13.40	0.08	−7.33	22.10	−13.62	−1.05	t	0.22	0.83
	AsI MM	−2.14	20.25	−8.02	3.74	−1.14	22.86	−7.78	5.50	t	−0.36	0.72
	AcI R	7.27	35.10	−2.92	17.46	13.12	29.05	4.68	21.55	t	−1.22	0.23
	AcI L	3.64	36.60	−6.98	14.27	6.53	32.85	−3.00	16.07	t	−0.69	0.49
	AcI total	4.96	32.97	−4.61	14.53	9.81	28.11	1.64	17.97	t	−1.18	0.24
	AsI SCM	−3.73	14.44	−7.84	0.37	−5.25	15.65	−9.70	−0.80	t	0.84	0.40
	AsI DA	1.27	12.96	−2.58	5.12	1.75	12.79	−2.05	5.55	t	−0.33	0.74
Clenching in the intercuspal position	AsI TA	−0.66	14.08	−4.66	3.34	−1.08	18.88	−6.45	4.29	z	0.01	0.99
	AsI MM	4.27	19.02	−1.25	9.79	2.59	16.85	−2.30	7.48	t	1.31	0.20
	AcI R	−1.36	23.45	−8.17	5.45	−1.82	24.93	−9.06	5.42	t	0.20	0.84
	AcI L	−5.58	28.77	−13.93	2.77	−4.93	30.31	−13.73	3.87	t	−0.30	0.77
	AcI total	−3.24	23.13	−9.96	3.48	−3.59	26.23	−11.20	4.03	t	0.16	0.87
	AsI SCM	1.99	18.50	−3.27	7.25	1.47	18.27	−3.72	6.66	t	0.41	0.69
	AsI DA	0.62	22.25	−5.98	7.23	3.04	16.56	−1.88	7.96	z	0.73	0.47
Clenching on dental cotton rollers	AsI TA	−2.33	14.44	−6.43	1.78	−2.79	15.99	−7.33	1.76	t	0.28	0.78
	AsI MM	−0.01	14.98	−4.35	4.34	0.42	15.12	−3.97	4.81	t	−0.43	0.67
	AcI R	15.58	22.06	9.17	21.98	13.64	22.26	7.18	20.10	t	1.65	0.11
	AcI L	13.17	23.13	6.46	19.89	10.07	25.08	2.79	17.35	t	2.02	0.05
	AcI total	14.58	20.10	8.75	20.42	11.82	21.36	5.61	18.02	t	2.35	0.02 * ES = 0.34
	AsI SCM	−0.12	18.15	−5.28	5.03	−2.22	17.05	−7.07	2.62	t	1.66	0.10
	AsI DA	0.50	19.80	−5.38	6.38	2.13	18.73	−3.44	7.69	t	−0.90	0.37
Maximum mouth opening	AsI TA	3.37	19.52	−2.17	8.92	2.89	19.09	−2.53	8.32	t	0.26	0.80
	AsI MM	3.98	15.49	−0.52	8.48	3.72	17.27	−1.29	8.74	t	0.15	0.88
	AcI R	12.56	23.32	5.78	19.33	12.87	23.92	5.92	19.81	t	−0.16	0.87
	AcI L	13.16	23.60	6.31	20.01	13.31	23.49	6.48	20.13	t	−0.06	0.95
	AcI total	12.68	21.22	6.52	18.84	13.23	21.94	6.86	19.60	t	−0.28	0.78
	AsI SCM	3.05	17.00	−1.78	7.88	2.28	16.75	−2.49	7.04	t	0.81	0.42
	AsI DA	0.43	13.50	−3.58	4.44	−0.84	12.73	−4.62	2.94	z	1.10	0.27

AcI—Activity index; AsI—Asymmetry Index; TA—the temporalis anterior; MM—the superficial part of the masseter muscle; SCM—the middle part of the sternocleidomastoid muscle; DA—the anterior belly of the digastric muscle; R—right site; L—left site; ES - Effect Size; * Significant difference.

**Table 2:** A comparison of the results of the functional indexes during two conditions (eyes open and closed) at rest, during clenching in the intercuspal position, clenching on dental cotton rollers, and maximum mouth opening.

	**Indices**	**Eyes Open**				**Eyes Closed**				**Statistics**		
		M	SD	95%CI		M	SD	95%CI		Test	Test Result	*p* Value
Clenching in the intercuspal position	FCI TA R	84.10	94.14	57.35	110.86	77.51	60.63	60.28	94.74	z	0.33	0.74
	FCI TA L	80.50	106.35	50.28	110.73	70.54	55.31	54.82	86.25	z	0.53	0.60
	FCI TA total	79.22	98.58	51.20	107.23	70.32	50.86	55.87	84.78	z	0.63	0.53
	FCSI TA	5.90	24.51	−1.06	12.87	5.90	27.21	−1.84	13.63	t	0.00	1.00
	FCI MM R	70.01	60.49	52.44	87.57	63.45	56.89	46.93	79.97	z	1.56	0.12
	FCI MM L	66.64	61.33	48.83	84.45	60.76	53.53	45.21	76.30	z	1.62	0.11
	FCI MM total	66.61	59.19	49.42	83.80	60.19	52.77	44.87	75.52	z	1.63	0.10
	FCSI MM	6.33	26.20	−1.28	13.94	3.36	27.03	−4.49	11.21	t	1.08	0.29
	FCAI R	−8.10	36.97	−18.83	2.64	−13.72	36.59	−24.35	−3.10	t	1.12	0.27
	FCAI L	−8.58	42.66	−20.97	3.81	−10.19	44.80	−23.20	2.82	t	0.40	0.69
	FCAI total	−7.89	36.26	−18.42	2.64	−12.45	39.11	−23.80	−1.09	t	1.09	0.28
	FCI SCM R	8.84	8.34	6.48	11.21	7.86	7.06	5.85	9.87	z	2.65	0.01 * ES = 0.37
	FCI SCM L	8.42	9.48	5.72	11.11	6.67	5.70	5.04	8.29	z	2.62	0.01* ES = 0.37
	FCI SCM total	8.48	8.53	6.06	10.90	7.09	5.92	5.41	8.78	z	2.37	0.02 * ES = 0.33
	FCSI SCM	5.60	22.41	−0.77	11.96	6.58	20.90	0.64	12.52	t	−0.49	0.63
	FCI DA R	12.33	9.50	9.51	15.15	11.96	10.45	8.86	15.06	z	0.99	0.32
	FCI DA L	13.68	14.12	9.49	17.88	11.79	11.97	8.24	15.34	z	1.61	0.11
	FCI DA total	13.00	10.44	9.90	16.10	11.85	11.08	8.56	15.13	z	1.47	0.14
	FCSI DA	−0.54	20.92	−6.75	5.67	1.37	16.04	−3.40	6.13	z	0.15	0.88
Clenching on dental cotton rollers	FCI TA R	77.01	87.88	52.04	101.99	76.71	53.22	61.59	91.84	z	1.45	0.15
	FCI TA L	73.55	100.15	45.09	102.01	69.47	46.86	56.16	82.79	z	1.69	0.09
	FCI TA total	72.54	92.84	46.15	98.92	69.33	44.14	56.79	81.88	z	1.60	0.11
	FCSI TA	4.18	24.07	−2.66	11.02	4.49	26.50	−3.04	12.02	t	−0.10	0.92
	FCI MM R	78.53	56.95	61.99	95.06	76.28	57.82	59.50	93.07	z	0.57	0.57
	FCI MM L	79.90	71.33	59.19	100.61	78.81	63.49	60.38	97.25	z	0.18	0.85
	FCI MM total	77.15	62.76	58.93	95.37	74.92	57.96	58.09	91.75	z	0.21	0.84
	FCSI MM	2.04	24.43	−5.06	9.13	1.20	27.11	−6.67	9.07	t	0.29	0.78
	FCAI R	7.46	37.49	−3.43	18.34	0.22	33.02	−9.37	9.80	t	1.58	0.12
	FCAI L	7.60	41.01	−4.31	19.51	2.50	42.05	−9.71	14.71	t	1.18	0.24
	FCAI total	8.22	36.39	−2.34	18.79	1.22	34.81	−8.88	11.33	t	1.71	0.09
	FCI SCM R	10.65	8.44	8.25	13.05	10.33	8.68	10.74	17.03	t	1.39	0.16
	FCI SCM L	10.03	8.17	7.71	12.35	9.47	6.60	10.27	18.21	t	0.78	0.44
	FCI SCM total	10.18	7.90	7.93	12.42	9.71	7.15	7.68	11.75	t	1.15	0.25
	FCSI SCM	3.44	21.22	−2.59	9.47	2.93	20.72	−2.96	8.82	t	0.23	0.82
	FCI DA R	14.02	8.48	11.50	16.54	13.88	10.59	10.74	17.03	z	0.80	0.42
	FCI DA L	13.82	7.87	11.49	16.16	14.24	13.38	10.27	18.21	z	0.88	0.38
	FCI DA total	13.89	7.77	11.58	16.20	14.06	11.77	10.57	17.56	z	0.75	0.45
	FCSI DA	−0.56	18.46	−6.04	4.92	0.49	17.53	−4.72	5.69	z	0.62	0.54
Maximum mouth opening	FOI TA R	4.66	4.72	3.32	6.00	4.46	3.75	3.39	5.52	z	0.29	0.77
	FOI TA L	3.58	3.45	2.60	4.56	3.91	3.71	2.85	4.96	z	5.26	<0.001 * ES = 0.74
	FOI TA total	3.91	3.67	2.87	4.95	3.99	3.38	3.03	4.95	z	1.16	0.24
	FCSI TA	9.58	26.93	1.92	17.23	9.57	28.32	1.52	17.62	t	0.00	1.00
	FOI MM R	6.01	9.96	3.11	8.90	6.01	11.00	2.82	9.20	z	1.06	0.29
	FOI MM L	4.98	6.90	2.98	6.99	4.75	6.07	2.98	6.51	z	0.39	0.70
	FOI MM total	5.29	7.95	2.98	7.60	5.11	7.72	2.86	7.35	z	0.88	0.38
	FOSI MM	5.87	23.30	−0.89	12.64	4.28	28.71	−4.06	12.61	t	0.44	0.66
	FOAI R	5.01	40.15	−6.65	16.67	−0.32	31.52	−9.48	8.83	t	1.03	0.31
	FOAII L	8.93	29.81	0.28	17.59	6.10	31.49	−3.05	15.24	t	0.70	0.49
	FOAI total	7.21	31.51	−1.93	16.36	2.99	27.01	−4.85	10.83	t	1.03	0.31
	FOI SCM R	11.19	12.63	7.60	14.78	11.19	12.46	7.65	14.73	z	1.26	0.21
	FOI SCM L	11.50	19.21	6.04	16.96	11.50	19.13	6.06	16.93	z	0.48	0.63
	FOI SCM total	11.10	15.30	6.75	15.45	11.05	15.30	6.71	15.40	z	1.10	0.27
	FOSI SCM	6.77	21.62	0.62	12.91	7.35	22.96	0.82	13.87	t	−0.29	0.77
	FOI DA R	48.47	34.95	38.09	58.85	44.34	26.76	36.40	52.29	z	0.89	0.37
	FOI DA L	47.54	31.64	38.14	56.93	45.57	25.21	38.08	53.05	z	0.29	0.77
	FOI DA total	47.61	32.38	37.99	57.23	44.54	25.23	37.04	52.03	z	0.61	0.54
	FOSI DA	−0.88	16.12	−5.67	3.91	−2.69	17.61	−7.92	2.54	t	1.11	0.27

FCI—Functional Clenching Index; FCAI—Functional Clenching Activity Index; FCSI—Functional Clenching Symmetry Index; FOI—Functional Opening Index; FOSI—Functional Opening Symmetry Index; FOAI—Functional Opening Activity Index; TA—the temporalis anterior; MM—the superficial part of the masseter muscle; SCM—the middle part of the sternocleidomastoid muscle; DA—the anterior belly of the digastric muscle; R—right site; L—left site; ES - Effect Size; * Significant difference.

## Data Availability

Not applicable.
